# Septic arthritis of the pubis symphysis: clinical and therapeutic features

**DOI:** 10.11604/pamj.2017.26.215.12204

**Published:** 2017-04-24

**Authors:** Zeineb Alaya, Houneida Zaghouani, Walid Osman, Lassad Hassini, Nader Naouar, Mohamed Laziz Ben Ayèche, Elyès Bouajina

**Affiliations:** 1 Department of Rheumatology, Farhat Hached Hospital, Faculty of Medicine of Sousse, Sousse, Tunisia; 2 Department of Radiology, Farhat Hached Hospital, Faculty of Medicine of Sousse, Sousse, Tunisia; 3 Department of Orthopaedics, Sahloul Hospital, Faculty of Medicine of Sousse, Sousse, Tunisia

**Keywords:** Infection, pubic symphysis, MRI, biopsy, antibiotics, surgery

## Abstract

Septic arthritis of the pubis symphysis is rare and difficult to diagnose. The objective of our study was to describe the biological, clinical, radiological and therapeutic aspects of this disease. This is a retrospective study of 4 cases of septic arthritis of the pubic symphysis collected in the Department of Rheumatology and Orthopaedics in Sousse in Tunisia over a period of 16 years (2000-2016). Our population consists of 3 women and one men. The mean age was 47 years (18-83). Clinical signs of appeal were inflammatory groin pain, pubic pain and fever. Symptoms appeared after forceps delivery in 2 cases, after surgery on the pelvis in one case and in a context of sepsis in one case. Radiographs showed pubic disjunction with irregular shoreline in all cases. CT performed in all patients and MRI in 2 patients showed erosions of the banks of the pubic symphysis with infiltration of the soft parts in all cases. The causative organisms were isolated in 3 cases by biopsy of soft tissue abscess under CT in 2 cases and vaginal swab in one case. Identified germs were staphylococcus aureus Méti-S (n=1), proteus mirabilis (n=1) and varied flora (n=1). The treatment consisted of appropriate antibiotics in all cases and surgical drainage of soft tissue abscess resistant to medical treatment in 2 cases. The outcome was favorable in all cases. Diagnosis of septic arthritis of the pubic symphysis is based on clinic supported by microbiologic culture results, image methods, and proteins augment during acute phase.

## Introduction

Septic arthritis or osteomyelitis of the pubis symphysis is a rare condition that occurs in less than 1% of cases of osteomyelitis [1-3]. It is often misdiagnosed due to the fact that the usual presenting symptoms are very nonspecific, thus delaying definitive treatment [1-3]. It should be suspected in patients with inflammatory groin pain, pubic pain and fever [1,2,4]. It is frequently associated with prior gynaecological/urological surgery or pelvic malignancy [1,5]. Radiographic signs can be delayed or undetected in certain modalities of radiological investigation. Therefore, the diagnosis can be missed and treatment delayed [1,4]. The objective of our study was to describe the biological, clinical, radiological and therapeutic aspects of septic arthritis of the pubic symphysis.

## Methods

This is a retrospective, descriptive study of 4 cases of septic arthritis of the pubic symphysis collected in the Department of Rheumatology and Orthopaedics in Sousse in Tunisia over a period of **1**6 years (2000-20**1**6). Demographic, clinical, microbiologic, treatment, and outcome data were collected from the medical record using a data collection fiched. The diagnosis of septic arthritis of the pubic symphysis was retained on clinical, biological and imaging evocative signs.

## Results

**Figure 1:**
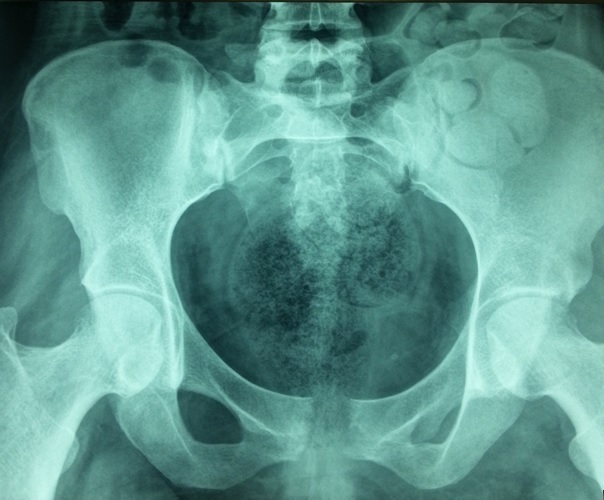
Radiography of the pelvis: disjunction of the pubic symphysis with erosions of the banks

**Figure 2:**
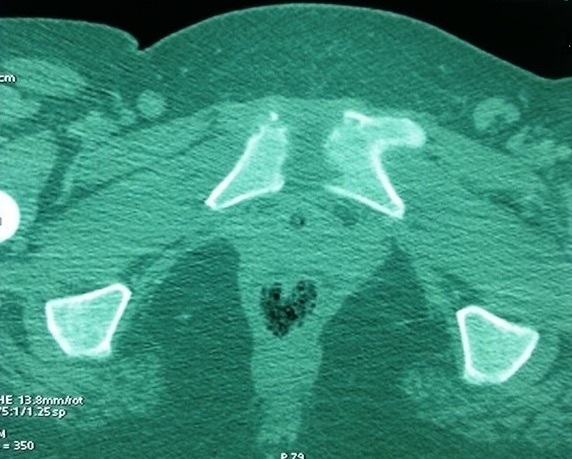
Scan of the pelvis: erosions of the banks of the pubic symphysis with infiltration of the soft parts

**Figure 3:**
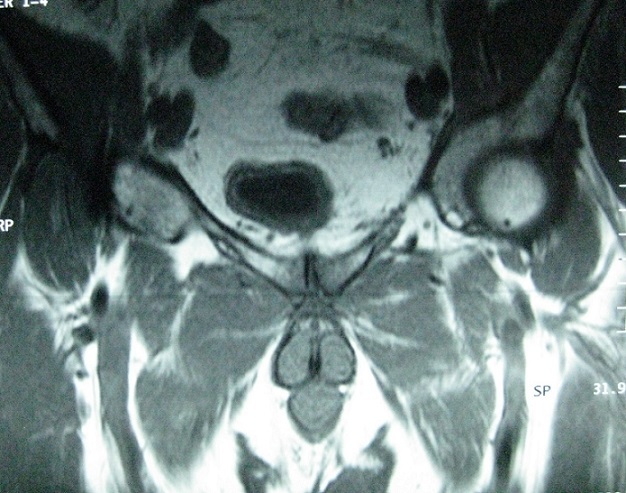
MRI of the pelvis: pubic symphysitis with inflammatory aspect of the major adductor muscles and thickening of the prepubic soft tissues

**Figure 4:**
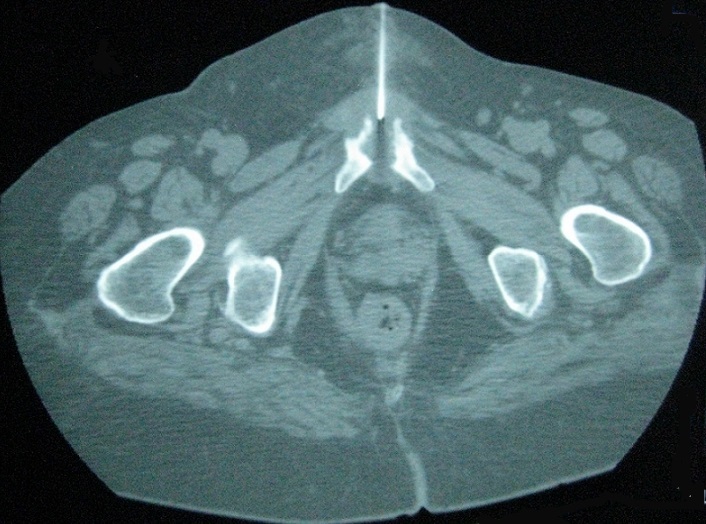
Puncture biopsy of the pubic symphysis under CT in a patient with an infectious pubic symphysitis

Our population consists of 3 women and one men. The mean age was 47 years (18-83). No comorbidity was recorded. Clinical signs of appeal were inflammatory groin pain, pubic pain and fever. Symptoms appeared 2 weeks after forceps delivery complicated by infectious endometritis in 2 cases, after surgery on the pelvis in one case and in a context of sepsis in one case. Walking was impossible in 2 cases. The mobilization of the hips and the pressure at the pubic symphysis were painful. The biological inflammatory syndrome was present in all cases. Plain radiographs showed pubic disjunction with irregular shoreline in all cases (Figure 1). CT performed in all patients (Figure 2) showed erosions of the banks of the pubic symphysis with infiltration of the soft parts. MRI of the pelvis performed in 2 patients (Figure 3), confirmed the pubic symphysite with an infiltration and abscess of the soft parts. A puncture biopsy of the pubic symphysis under CT was performed in all patients (Figure 4). The causative organisms were isolated in 3 cases by biopsy of soft tissue abscess in 2 cases and vaginal swab in one case. Identified germs were staphylococcus aureus Méti-S (n=1), proteus mirabilis (n=1) and varied flora (n=1). The treatment consisted of appropriate antibiotics in all cases and surgical drainage of soft tissue abscess resistant to medical treatment in 2 cases. The outcome was favorable in all cases.

## Discussion

Septic arthritis of the pubic symphysis, so called osteomyelitis pubis is the infection which involves pubic symphysis and its joint [1]. It is usually associated with pelvic surgery, pelvic malignancies, pregnancy, intravenous drug use and recent athletic activity [1,2,5-9]. This disease is not specific to any age group and can range from 7 to 86 years of age [7]. The diagnosis of osteomyelitis pubis is often missed or delayed due to the infrequency of the disease and its variable presentation [10]. Most common presenting signs and symptoms include fever, pubic tenderness, antalgic gait, and pain with active/passive range of motion of hip [1,3,7]. Insidious symptoms often delay the diagnosis; therefore, clinicians should consider this entity in patients presenting with pubic, groin or abdominal pain that increases on ambulation, and acute onset of fever [2]. The most common pathogen causing infections of pubis symphysis was found to be Staphylococcus aureus; however, Pseudomonas aeruginosa, Escherichia coli, Enterococcus sp., Mycobacterium tuberculosis, Salmonella sp., and Streptococcus sp., as well as others have also been reported in literature [1-3,7]. Infection of the symphysis pubis and non-infectious inflammation of the same joint, or osteitis pubis, are distinct entities that present similarly [10].

Septic arthritis of the pubic symphisis is distinguished from osteitis pubis by positive cultures [3]. Diagnosis is based on clinic supported by microbiologic culture results, image methods, and proteins augment during acute phase [1]. Laboratory values were not always abnormal, as in the study of Ross and Hu leukocytosis was observed in only 35% of patients [7]. ESR and CRP may be abnormal but are nonspecific [7]. Bacteremia, not a useful marker in the ED, was present in 73% of patients in the same study with blood culture results reported. Cultures of needle aspirates of the symphysis pubis were more sensitive with 86% positive in the same study [7]. CT and MRI examinations are essential to substantiate the diagnosis or to guide sampling [11]. Despite MRI being the most sensitive imaging test, only aspiration (ie, microorganism isolation) provides the ultimate proof of the presence of infection [4]. CT scan of the pelvis showed mild widening and erosive changes involving the pubic symphyis associated to fluid collection [5]. Microbiology cultures from an ultrasound or scan guided aspiration of the fluid collection reveals the germ [5].

The different diagnosis of osteolytic, destructive, and inflammatory processes around the symphysis are infectious osteitis pubis, inflammatory osteitis pubis, posttraumatic benign pubic osteolyses in elderly women, and malignant neoplasia [12]. Accurate diagnosis can be a challenge and requires a methodical approach and the use of a variety of diagnostic measures [12,13]. The antibiotic treatment is adjusted depending on the microbiological diagnosis, adding NSAIDs, and bed rest [1,7]. The duration of antibiotic therapy is on average 6 weeks [5,11]. Despite long-course intravenous antibiotherapy, >50% of cases require surgical debridement [7,14]. When adequate treatment is instituted, most individuals recover completely [1,4,7,11]. The emergency physician can make a difference in the course of the disease by recognizing the condition early, and starting the patient on the road to definitive workup and treatment, which involves pain control and long-term intravenous (IV) antibiotic therapy [10].

## Conclusion

Septic arthritis of the pubic symphysis is a rare cause of pubic and hip pain. His diagnosis is often missed or delayed due to the infrequency of the disease and its variable presentation. It should be suspected in patients with inflammatory groin pain, pubic pain and fever especially after delivery and pelvic surgery. In front of an osteolytic processes around the symphysis, the search for an infectious cause is paramount. Diagnosis is based on clinic supported by microbiologic culture results, image methods, and proteins augment during acute phase. MRI is essential for diagnosis. Long delays between the symptom onset and diagnosis are frequent and therefore awareness is paramount for early case detection. Long-course antibiotherapy is required and, in some cases, may preclude the need for surgical debridement.

### What is known about this topic

- Septic arthritis of the pubis symphysis is rare and difficult to diagnose;
- It follows in most cases pelvis surgery or delivery;
- The treatment is based on antibiotherapy.

### What this study adds

- clinical features of Septic arthritis of the pubis symphysis in the region of the center of Tunisia;
- benefits of biology and imaging in the diagnosis of Septic arthritis of the pubis symphysis;
- The progression is favorable if the diagnosis is early and antibiotherapy is adapted.
